# Peripheral inflammation increases PKR activation, Tau phosphorylation and amyloid β production in wild-type mice

**DOI:** 10.1186/1750-1326-8-S1-P32

**Published:** 2013-09-13

**Authors:** François Mouton-Liger, Anne-Sophie Rebillat, Clarisse Pace, Sarah Gourmaud, Mariko Taga, Claire Paquet, Jacques Hugon

**Affiliations:** 1 Inserm UMR-S839, Paris, France; 2 Hopital Lariboisière APHP, Paris, France; 3 University of Southampton, Southampton, UK

## Background

Systemic inflammation is correlated with dementia progression. Pro-inflammatory molecules can communicate from the periphery to the central nervous system to induce neuroinflammation and neurodegeneration. Our protein of interest is the pro-apoptotic kinase PKR (the double strand-RNA dependent protein kinase). Increased activated PKR levels were found in AD patients brain and cerebrospinal fluid. PKR activation can be triggered by inflammatory stresses and induces neurotoxicity *in vitro.* Is *in vivo* PKR-mediated inflammation involved in AD neurodegenerative process?

## Learning objective

To investigate whether PKR-mediated neuroinflammation could play a role in AD neurodegenerative process.

## Methods

C57BL/6 wild type mice were injected intraperitoneally with LPS (1mk/kg) versus saline once a day for 3 days to induce PKR activation and neuroinflammation (LPS is the bacilli gram negative endotoxin lipopolysaccharide).

Brains were collected and dissected; immunohistochemistry and western blotting were performed for neuroinflammation, PKR activation and AD pathological hallmarks (as Tau hyperphosphorylation).

## Results

Mice showed endotoxin-induced sickness behaviour includingbody weight loss and elevated serum cytokine levels.

Microglial activation, neuronal apoptosis, increase of PKR, GSK3β and Tau phosphorylation and amyloid β production were found in hippocampus and cortex of LPS-treated mice.

## Conclusions

PKR could be involved in the signalling of neurofibrillary tangles formation after a systemic inflammatory challenge.

